# Socioeconomic gradient in mortality of working age and older adults with multiple long-term conditions in England and Ontario, Canada

**DOI:** 10.1186/s12889-023-15370-y

**Published:** 2023-03-11

**Authors:** Anne Alarilla, Luke Mondor, Hannah Knight, Jay Hughes, Anna Pefoyo Koné, Walter P. Wodchis, Mai Stafford

**Affiliations:** 1 The Health Foundation, 8 Salisbury Square, London, UK; 2 ICES, Toronto, ON M4N 3M5 Canada; 3 Health System Performance Network, Toronto, ON Canada; 4 Department of Health Sciences, Lakehead University, Thunder Bay, ON Canada; 5 Institute of Health Policy Management & Evaluation, University of Toronto, Toronto, ON Canada; 6 Institute for Better Health, Trillium Health Partners, Mississauga, ON Canada

**Keywords:** Multimorbidity, Inequalities, Survival, Primary health care

## Abstract

**Background:**

There is currently mixed evidence on the influence of long-term conditions and deprivation on mortality. We aimed to explore whether number of long-term conditions contribute to socioeconomic inequalities in mortality, whether the influence of number of conditions on mortality is consistent across socioeconomic groups and whether these associations vary by working age (18–64 years) and older adults (65 + years). We provide a cross-jurisdiction comparison between England and Ontario, by replicating the analysis using comparable representative datasets.

**Methods:**

Participants were randomly selected from Clinical Practice Research Datalink in England and health administrative data in Ontario. They were followed from 1 January 2015 to 31 December 2019 or death or deregistration. Number of conditions was counted at baseline. Deprivation was measured according to the participant’s area of residence. Cox regression models were used to estimate hazards of mortality by number of conditions, deprivation and their interaction, with adjustment for age and sex and stratified between working age and older adults in England (*N* = 599,487) and Ontario (*N* = 594,546).

**Findings:**

There is a deprivation gradient in mortality between those living in the most deprived areas compared to the least deprived areas in England and Ontario. Number of conditions at baseline was associated with increasing mortality. The association was stronger in working age compared with older adults respectively in England (HR = 1.60, 95% CI 1.56,1.64 and HR = 1.26, 95% CI 1.25,1.27) and Ontario (HR = 1.69, 95% CI 1.66,1.72 and HR = 1.39, 95% CI 1.38,1.40). Number of conditions moderated the socioeconomic gradient in mortality: a shallower gradient was seen for persons with more long-term conditions.

**Conclusions:**

Number of conditions contributes to higher mortality rate and socioeconomic inequalities in mortality in England and Ontario. Current health care systems are fragmented and do not compensate for socioeconomic disadvantages, contributing to poor outcomes particularly for those managing multiple long-term conditions. Further work should identify how health systems can better support patients and clinicians who are working to prevent the development and improve the management of multiple long-term conditions, especially for individuals living in socioeconomically deprived areas.

**Supplementary Information:**

The online version contains supplementary material available at 10.1186/s12889-023-15370-y.

## Background

The prevalence of multiple long-term conditions (known as multimorbidity) is rising [1, 2], and in the general population varies between 13 to 72% depending on the definition, country, data source, and age range [3]. Multiple long-term condition prevalence is increasing in most demographic groups and is higher in socioeconomically deprived groups [4–11]. Greater socioeconomic deprivation can increase the exposure to health-damaging factors leading to higher vulnerability, which may lead to higher prevalence and earlier onset of multiple-long term conditions among deprived groups [2, 6, 7, 10].

Multiple long-term conditions also influence subsequent outcomes (including mortality/survival), and it is possible that the intersection of multiple long-term conditions and socioeconomic factors may contribute to worse outcomes. People with multiple-long term conditions are at risk of poorer outcomes such as lower quality of life and higher mortality rate [12–16]. Managing multiple-long term conditions is also associated with higher health care use with direct and indirect costs such as increasing inability to work [17, 18]. There is evidence that socioeconomic deprivation contributes to higher burden of treatment associated with managing multiple long-term conditions [19–21] including potential issues in treatment or care quality (e.g. polypharmacy, drug adverse events, compliance) [22–26]. However, it is less clear whether socioeconomic deprivation also contributes to poorer survival for those with multiple long-term conditions. Current evidence is mixed on whether socioeconomic deprivation exacerbates the mortality risk of people with multiple long-term conditions [13, 27–32] It is important to understand these socioeconomic inequalities in order to mitigate adverse health outcomes associated with multiple long-term conditions.

In addition, even though the socioeconomic gradients in morbidity and mortality are well recognised [13, 27–36], there has been less attention to whether the association between multiple long-term conditions and mortality holds across age groups. A focus on working age adults may be warranted because the association between socioeconomic deprivation and mortality is stronger at younger ages and socioeconomic inequalities in mortality have widened over time in people of working age [36–38]. Similarly, socioeconomic inequalities in multiple long-term condition prevalence have increased over time for all ages but increases are greatest in people of working age [2]. Emerging evidence suggests that the influence of number of conditions on survival weakens with age and is stronger for working age adults (under 65 s) than older adults (65 + years), though this warrants replication in nationally representative samples [13]. Together these studies suggest that socioeconomic deprivation, having multiple long-term conditions, and plausibly the combination of these could have greater impact on survival in people of working age compared with older people, though this has not been directly tested.

Drawing conclusions from these different studies is difficult because they used different measures of multiple long-term conditions (including the number and type of conditions) and because national or local differences in inequalities in health and access to healthcare could lead to differences in outcomes. Our study will provide a cross-jurisdiction comparison between England and Ontario (Canada’s largest province by population). Both jurisdictions offer universal healthcare and have comparable measures of socioeconomic deprivation (at the area level). They have also both previously prioritised tackling inequalities in primary care but have taken different approaches to do so which has resulted in differing trajectories in inequalities in mortality [39].

Consequently, the aims of this retrospective cohort study are to: i) explore whether number of conditions contribute to explaining socioeconomic inequalities in mortality, ii) examine whether the association between number of conditions and mortality differs by level of socioeconomic deprivation and iii) assess whether the magnitude or direction of these associations vary between working age and older adults. We replicate the analysis using the same long-term health conditions and comparable data sets from England and Ontario. We use individual-level measures of long-term conditions, age, sex and rely on area-level measures of socioeconomic deprivation. Both individual level socioeconomic position and area level socioeconomic deprivation indicators have been used to study the gradient in multiple long-term conditions [4–11]. Although multilevel study designs have shown that each can independently affect outcomes such as mortality through multiple material, psychosocial and behavioural mechanisms, area level deprivation is often recommended for use as a proxy for patient socioeconomic deprivation in population-based studies using routine health data [40].

## Methods

### Participants

England

A simple, random sample of 600,000 adults aged 18 + was drawn from the Clinical Practice Research Datalink (CPRD Aurum). This is an ongoing pseudonymised research database from routinely collected primary care records. Over 98% of the population in England is registered in a primary care practice and CPRD Aurum is derived from the largest of the four IT systems used across primary care practices in England (56% of practices) [41]. It is representative of the English population in terms of geographical spread, deprivation, age and gender [41] and includes primary care records from over 40 million patients (13.35 million patients currently registered as of February 2022). Primary care records include information on diagnoses, symptoms, prescriptions, referrals, and tests. Eligible adults (*n* = 8,750,306) were registered in a CPRD practice on 1st January 2014 (to ensure records were up to date at least one year before the study start), were alive and still registered at the study start on 1st January 2015, and were eligible for linkage to Hospital Episode Statistics (HES) and Office for National Statistics mortality data. They were followed until the study end (31 December 2019) or death if this was earlier and were censored if they left the CPRD practice or the practice stopped providing data to CPRD. The CPRD reviewed and approved the ethics and methods of this study (eRAP protocol number 20_000239). Informed individual consent was not required as patients are pseudonymised and cannot be identified from CPRD. Patients were still able to opt-out from sharing their data for research purposes through the national data opt-out scheme introduced in 2018 [42].

Ontario, Canada.

A simple, random sample of adults was drawn from health administrative data of residents for the Ontario population eligible for universal health coverage (Supplementary Table 1). These data are linked using unique encoded identifiers and analysed at ICES. Specifically, we identified all persons in the Registered Persons Database who were alive and residing in the province of Ontario between the ages of 18 and 105 years on January 1, 2015 (*n* = 11,916,158). Residents were excluded from the study if they did not have a valid OHIP health card at index, or for the full 365 days prior to index (n = 1,188,678). From those remaining (*n* = 10,727,480) we retained a random sample of 600,000 for analysis. Participants were followed up until the study end (31 December 2019) or death if this was earlier and individuals where no outcome was observed were censored at the study end date or the date at which they were no longer eligible for OHIP coverage, whichever came first. The use of data in this project was authorized under section 45 of Ontario’s Personal Health Information Protection Act, which does not require review by a Research Ethics Board. Informed consent from participants was not required because we used health information routinely collected in Ontario and held in health administrative databases.

### Measures

Survival time was calculated from 1st January 2015 to death or censoring. Age (in years) and sex (classed into two categories (men/women) were measured at baseline.

Socioeconomic deprivation was captured by area level deprivation. In England this was using 2015 Index of Multiple Deprivation (IMD) decile in the patient’s area of residence based on lower-level super output area boundaries and includes dimensions of education, living environment, income, employment, housing, health and disability and crime [43]. In Ontario, deprivation was measured using the 2016 material deprivation index (ON-MARG data). This area-based measure includes dimensions of income, education, housing and family structure characteristics and is closely connected to poverty [44]. For each measure decile 1 represents the 10^th^ least deprived areas and decile 10 represents the 10^th^ most deprived areas for analyses. Supplementary Table 2 describes the components of IMD and ON-Marg indices. These are comparable indicators of socioeconomic deprivation that have been previously used to compare socioeconomic inequalities in mortality across these jurisdictions [39].

The number of long-term conditions was counted at study start. In the primary analyses, we used a sub-set of nineteen physical and mental health conditions that have previously been associated with higher mortality risk, poorer functioning, and requiring primary care input [8, 9]. To ensure that our analyses between the two jurisdictions are comparable, long-term conditions included need to be measurable both in the England and Ontario samples. These were alcohol misuse, arthritis, asthma, atrial fibrillation, cancer (any), chronic obstructive pulmonary disease, congestive heart failure, dementia, diabetes, epilepsy, ischemic heart disease, hypertension, kidney disease, multiple sclerosis, Parkinson’s disease, psoriasis, schizophrenia, stroke (including transient ischemic attack) and thyroid disorder. To test whether the total number and type of conditions included influenced the relationship with mortality, we also included an additional seven conditions in the sensitivity analyses including mental health conditions. These were anxiety and depression, blindness, bronchiectasis, diverticulosis, hearing loss, liver disease and substance misuse. These were limited to sensitivity analyses because there was an established SNOMED code for England, but the constructs did not align well, or we were unable to check our prevalence estimate with published data for Ontario (Supplementary Table 3 for England and Supplementary Table 4 for Ontario). In England, conditions were coded using previously published SNOMED code list [2, 45] and/or prescription code from CPRD (see Supplementary Table 3). For Ontario, conditions were identified using a mix of disease-specific registries using validated algorithms for health administrative data, and from OHIP, DAD, OMHRS and NACRS datasets using case ascertainment algorithms from the Canadian Chronic Disease Surveillance system [46, 47] or code sets identified from prior research studies (see Supplementary Table 4). For each person in the dataset, we summed the total number of conditions prevalent at baseline.

### Statistical analyses

The final analytical sample (England: *N* = 599,487, Ontario: *N* = 594,546) included those with complete data on baseline sex, age and deprivation (England: *N* = 513 excluded, Ontario: *N* = 5,474 excluded). Excluded patients in England and Ontario are more likely to be younger and male and in Ontario they had more long-term conditions (Supplementary Table 5).

The association between survival time and deprivation was modelled using Cox proportional hazards models. It was not possible to merge the two patient-level data sets so separate analyses were done for England and Ontario but the same modelling approach was used. For England, two-level models were used to allow for the clustering of patients within GP practices. The assumption of proportional hazard was tested statistically and visually using Schoenfeld residuals in a model that includes, age, sex, number of long-term conditions and deprivation. For England, the results show varying baseline hazard for age (*p* < 0.001) and sex (*p* = 0.060) at later follow up times. For Ontario, the results show varying baseline hazard for age (*p* < 0.001) and number of conditions (*p* < 0.001) at later follow up times. However, when these variables were included as time-varying for the respective models in both jurisdictions, the estimates did not materially change and therefore the simpler models are presented and were deemed to be representative.

Model 1 included age, sex and deprivation. In model 2, we included deprivation and the interaction of age by deprivation and compared with model 1 to test the hypothesis that the association between deprivation and mortality differs with age. If the interaction was significant then subsequent models were stratified by age groups, to allow for the relationship between deprivation and mortality to differ between working age (18–64 years) and older (65 + years) adults.

To examine whether number of long-term conditions contributed to the association between mortality and deprivation, we added number of long-term conditions in the model (model 3) [48]. Number of long-term conditions was added as a continuous variable after confirming the linear association with survival time (Supplementary Fig. 1). Number of long-term conditions was capped at 6 conditions as non-linearity in survival was observed for persons with more than 6 conditions which was likely affected by smaller and variable sample sizes in these groups.

To test whether the association between mortality hazard and number of conditions was consistent across deprivation groups, interaction terms for deprivation by number of conditions were added to the stratified models (model 4).

To assess the improvement in goodness of fit, likelihood ratio tests were performed for nested models. Significance refers to statistical significance at 5% level.

In sensitivity analyses, models 3 and 4 were repeated for England and Ontario to include this list of 26 conditions.

All statistical analyses were conducted using R software for England and using SAS (data management) and Stata (analysis) for Ontario, Canada.

## Results

At the end of the follow up period, 32,804 (5.5%) participants had died in England and 27,947 (4.7%) participants had died in Ontario (Table 1). A higher proportion of participants was censored during the follow up period in England than in Ontario (Table 1), those censored in England were more likely to be working age and had fewer long-term conditions compared with the sample with full follow-up time, but they were spread evenly across deprivation deciles (Supplementary Table 6). Those living in the most deprived areas in England were more likely to be younger compared to patients living in the least deprived areas, whereas in Ontario, patients living in the most deprived tenth of areas were more likely to be either younger (18–29 years) or older (80 + years) (Table 2).

**Table 1:** Characteristics of analytical sample in England and Ontario (Canada)

	England	Canada (Ontario)
N	599,487	594,526
Baseline age N (%)		
Working age adults (18–64 years)	462,761 (77.2%)	474,824 (79.9%)
Older adults (65 + years)	136,726 (22.8%)	119,702 (20.1%)
Women N (%)	300,098 (50.1%)	305,309 (51.4%)
Deprivation Decile		
1- Least Deprived	67,235 (11.2%)	64,575 (10.9%)
2	63,421 (10.6%)	66,409 (11.2%)
3	63,803 (10.6%)	62,172 (10.5%)
4	60,109 (10.0%)	61,854 (10.4%)
5	57,615 (9.6%)	56,641 (9.5%)
6	58,714 (9.8%)	58,272 (9.8%)
7	61,846 (10.3%)	55,394 (9.3%)
8	56,833 (9.5%)	56,865 (9.6%)
9	59,020 (9.8%)	54,743 (9.2%)
10- Most Deprived	50,891 (8.5%)	57,601 (9.7%)
Died during follow-up N (%)	32,804 (5.5%)	27,947 (4.7%)
Censored during follow up N (%)	139,124 (23.2%)	9,312 (1.6%)
Baseline number of long-term conditions mean (sd)	0.70 (1.11)	0.90 (1.31)

**Table 2:** Characteristics of analytical sample by deprivation deciles in England and Ontario (Canada)

Deprivation	N	Working age adults (18–64 years)	Older adults (65 + years)	Women N (%)	Died during follow-up N (%)	Baseline number of long-term conditions mean (sd)
England (IMD)						
1 – Least Deprived	67,235	49,212 (73.2%)	18,023 (26.8%)	34,264 (51.0%)	3,365 (5.0%)	0.67 (1.05)
2	63,421	46,413 (73.2%)	17,008 (26.8%)	32,114 (50.6%)	3,519 (5.5%)	0.70 (1.09)
3	63,803	47,275 (74.1%)	16,528 (25.9%)	32,122 (50.3%)	3,618 (5.7%)	0.71 (1.11)
4	60,109	44,871 (74.6%)	15,238 (25.4%)	30,036 (50.0%)	3,381 (5.6%)	0.70 (1.10)
5	57,615	43,579 (75.6%)	14,036 (24.4%)	28,749 (49.9%)	3,193 (5.5%)	0.71 (1.11)
6	58,714	45,292 (77.1%)	13,422 (22.9%)	29,570 (50.4%)	3,198 (5.4%)	0.71 (1.13)
7	61,846	48,712 (78.8%)	13,134 (21.2%)	30,896 (50.0%)	3,406 (5.5%)	0.69 (1.12)
8	56,833	46,443 (81.7%)	10,390 (18.3%)	28,139 (49.5%)	2,917 (5.1%)	0.67 (1.11)
9	59,020	48,856 (82.8%)	10,164 (17.2%)	29,226 (49.5%)	3,142 (5.3%)	0.69 (1.13)
10- Most Deprived	50,891	42,108 (82.7%)	8,783 (17.3%)	24,982 (49.1%)	3,065 (6.0%)	0.74 (1.18)
Total	599,487	462,761 (77.2%)	136,726 (22.8%)	300,098 (50.1%)	32,804 (5.5%)	0.70 (1.11)
Canada (Ontario) (Material Deprivation)						
1 – Least Deprived	64,575	52,164 (80.8%)	12,411 (19.2%)	32,887 (50.9%)	2,454 (3.8%)	0.77 (1.20)
2	66,409	53,564 (80.7%)	12,845 (19.3%)	34,055 (51.3%)	2,509 (3.8%)	0.81 (1.23)
3	62,172	50,173 (80.7%)	11,999 (19.3%)	31,780 (51.1%)	2,397 (3.9%)	0.83 (1.23)
4	61,854	49,414 (79.9%)	12,440 (20.1%)	31,709 (51.3%)	2,740 (4.4%)	0.87 (1.28)
5	56,641	45,012 (79.5%)	11,629 (20.5%)	29,158 (51.5%)	2,550 (4.5%)	0.89 (1.28)
6	58,272	46,238 (79.3%)	12,034 (20.7%)	29,906 (51.3%)	2,784 (4.8%)	0.93 (1.33)
7	55,394	43,774 (79.0%)	11,620 (21.0%)	28,422 (51.3%)	2,813 (5.1%)	0.95 (1.34)
8	56,865	44,435 (78.1%)	12,430 (21.9%)	29,499 (51.9%)	3,105 (5.5%)	0.99 (1.39)
9	54,743	43,141 (78.8%)	11,602 (21.2%)	28,083 (51.3%)	3,280 (6.0%)	1.01 (1.42)
10- Most Deprived	57,601	46,909 (81.4%)	10,692 (18.6%)	29,810 (51.8%)	3,315 (5.8%)	1.02 (1.43)
Total	594,526	474,824 (79.9%)	119,702 (20.1%)	305,309 (51.4%)	27,947 (4.7%)	0.90 (1.31)

The unadjusted mean number of long-term conditions at baseline was lower in England (mean = 0.70, standard deviation = 1.11) than Ontario (mean = 0.90, standard deviation = 1.31) (Table 1). For both jurisdictions, those living in most deprived areas had a higher mean number of long-term conditions compared to those living in the least deprived areas (Table 2). The distributions of number of conditions by age group in England and Ontario (Canada) are similar (Supplementary Fig. 2) and prevalence of conditions for each jurisdiction is shown in a supplementary table (Supplementary Table 7).

**Fig. 1:**
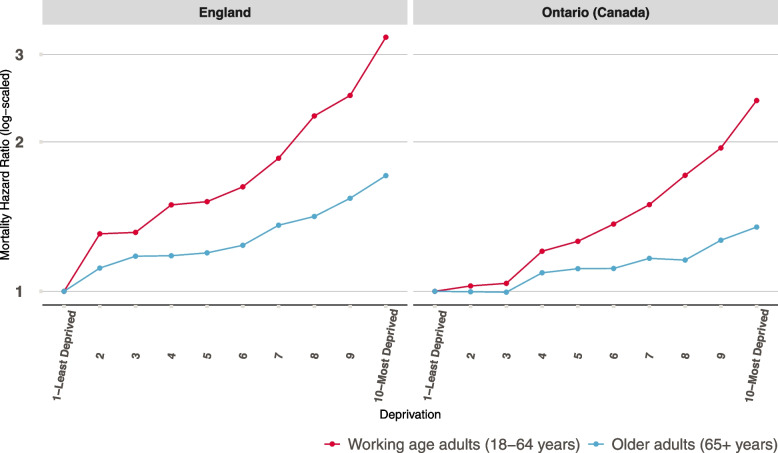
Mortality Hazard Ratio (log-scaled) by deprivation for the two age groups in England and Ontario (Canada) ^a^. Estimates are shown for persons with no condition. Mortality hazard ratio greater than 1 means a higher mortality rate during follow up compared to a person living in the least deprived areas (decile 1) from the same age group in the same jurisdiction. ^a^ Based on a model that includes linear age, sex and deprivation stratified by age groups

We found a statistically significant interaction between age and deprivation in England and Ontario (supplementary table 8 for full estimates). Age-stratified models show that the deprivation gradient in mortality rate was steeper in working age adults than older adults; this is consistent for England and Ontario (Fig. 1).

For example, in England, those living in the most deprived areas (decile 10) had a higher mortality rate compared to their counterparts living in the least deprived areas (decile 1), this was evident for working age adults and older adults with hazard ratios (HR) of 3.25 (95% CI 2.88, 3.66; Table 3 for full estimates) and 1.71 (95% CI 1.61,1.81; Table 4 for full estimates), respectively. In Ontario, for working age and older adults, those living in the most deprived areas (decile 10) had a higher mortality rate compared to those living in the least deprived areas (decile 1), with HRs of 2.42 (95% CI 2.17, 2,71; Table 3 for full estimates) and 1.35 (95% CI 1.27, 1.43; Table 4 for full estimates), respectively. For both age groups, the deprivation gradient was steeper in England than in Ontario (Fig. 1).

**Table 3:** Hazard Ratios (HR) and Confidence Intervals (CI) of model 2 and 3 for working age adults (18–64 years) in England and Ontario (Canada)

		England				Ontario			
		Model 2		Model 3		Model 2		Model 3	
Covariate	Reference Group	HR	95% CI	HR	95% CI	HR	95% CI	HR	95% CI
Age	per 1 year increase	1.09	1.09–1.09	1.07	1.07–1.07	1.08	1.08–1.09	1.06	1.05–1.06
Women	Men	0.70	0.66–0.74	0.69	0.66–0.73	0.61	0.58–0.64	0.61	0.58–0.64
Deprivation	1- Least Deprived								
2		1.31	1.14–1.49	1.27	1.11–1.46	1.03	0.90–1.16	0.99	0.88–1.13
3		1.31	1.15–1.50	1.26	1.11–1.44	1.04	0.91–1.18	0.99	0.87–1.13
4		1.49	1.31–1.70	1.42	1.25–1.62	1.20	1.06–1.36	1.13	1.00–1.28
5		1.52	1.33–1.73	1.42	1.24–1.62	1.26	1.11–1.43	1.17	1.03–1.32
6		1.62	1.43–1.85	1.47	1.29–1.67	1.37	1.21–1.54	1.20	1.06–1.36
7		1.85	1.63–2.11	1.67	1.46–1.90	1.49	1.32–1.69	1.29	1.14–1.46
8		2.26	1.99–2.55	1.94	1.72–2.19	1.71	1.52–1.93	1.44	1.28–1.61
9		2.48	2.19–2.91	2.08	1.84–2.35	1.94	1.74–2.18	1.59	1.42–1.79
10- Most Deprived		3.25	2.88–3.66	2.64	2.34–2.98	2.42	2.17–2.71	1.83	1.64–2.05
Baseline number of conditions	per 1 condition increase			1.60	1.56–1.64			1.69	1.66–1.72
Likelihood ratio test between Model 2 and 3		*X*^*2*^(1) = 1486.90, *p* < 0.001				*X*^*2*^(1) = 3186.19, *p* < 0.001

**Table 4:** Hazard Ratios (HR) and Confidence Intervals (CI) of model 2 and 3 for older adults (65 + years) in England and Ontario (Canada)

		England				Ontario			
		Model 2		Model 3		Model 2		Model 3	
Covariate	Reference Group	HR	95% CI	HR	95% CI	HR	95% CI	HR	95% CI
Age	per 1 year increase	1.13	1.13–1.13	1.11	1.11–1.12	1.12	1.12–1.12	1.10	1.10–1.10
Women	Men	0.76	0.74–0.78	0.78	0.76–0.80	0.72	0.70–0.73	0.76	0.74–0.79
Deprivation	1- Least Deprived								
2		1.11	1.06–1.17	1.09	1.03–1.14	1.00	0.94–1.06	0.98	0.92–1.04
3		1.18	1.12–1.24	1.13	1.08–1.19	1.00	0.94–1.06	0.98	0.92–1.04
4		1.18	1.12–1.25	1.14	1.08–1.20	1.09	1.03–1.16	1.05	0.99–1.11
5		1.20	1.13–1.26	1.14	1.09–1.21	1.11	1.04–1.18	1.08	1.01–1.15
6		1.24	1.17–1.31	1.16	1.1–1.23	1.11	1.05–1.18	1.05	0.99–1.12
7		1.36	1.29–1.43	1.27	1.2–1.34	1.17	1.10–1.24	1.11	1.05–1.18
8		1.42	1.34–1.5	1.30	1.23–1.38	1.16	1.09–1.23	1.10	1.04–1.17
9		1.54	1.46–1.63	1.39	1.32–1.47	1.27	1.19–1.34	1.17	1.11–1.24
10 -Most Deprived		1.71	1.61–1.81	1.51	1.43–1.60	1.35	1.27–1.43	1.21	1.14–1.29
Baseline number of conditions	per 1 condition increase			1.26	1.43–1.70			1.39	1.38–1.40
Likelihood ratio test between Model 2 and 3		*X*^*2*^(1) = 3643.10, *p* < 0.001				*X*^*2*^(1) = 6475.13, *p* < 0.001			

Number of long-term conditions was associated with increased mortality rate; this was stronger for working age adults (Table 3) than older adults (Table 4). Working age adults had a higher mortality rate (HR = 1.60, 95% CI 1.56,1.64) associated with an increase of one condition than older adults (HR = 1.26, 95% CI 1.25,1.27) in England. Similarly, in Ontario, the mortality hazard ratio associated with increasing number of conditions was higher for working age adults (HR = 1.69, 95% CI 1.66,1.72) than older adults (HR = 1.39, 95% CI 1.38,1.40). After accounting for number of long-term conditions, the association between deprivation and mortality decreased in magnitude but remained substantial and significant (Tables 3 and 4 for full estimates).

**Fig. 2:**
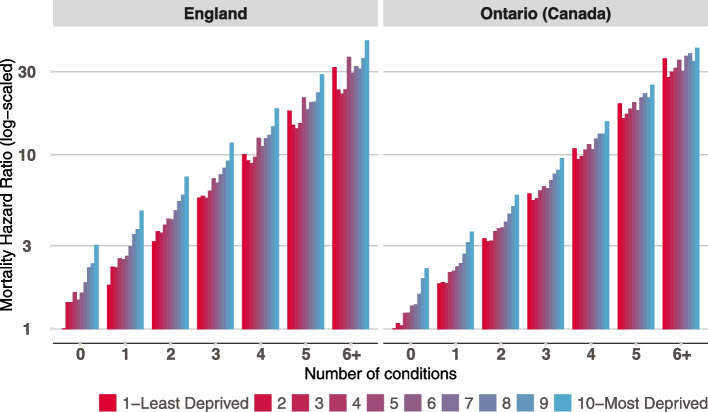
Mortality Hazard Ratio (log scaled) by deprivation and number of conditions for working adults (18–64 years) in England and Ontario (Canada). Estimates are shown based a model with sex, deprivation, number of long-term conditions and interaction between deprivation and number of conditions. Mortality hazard ratio of greater than 1 means a higher likelihood of dying during follow up compared to a person from the least deprived areas (decile 1) and no conditions

**Fig. 3:**
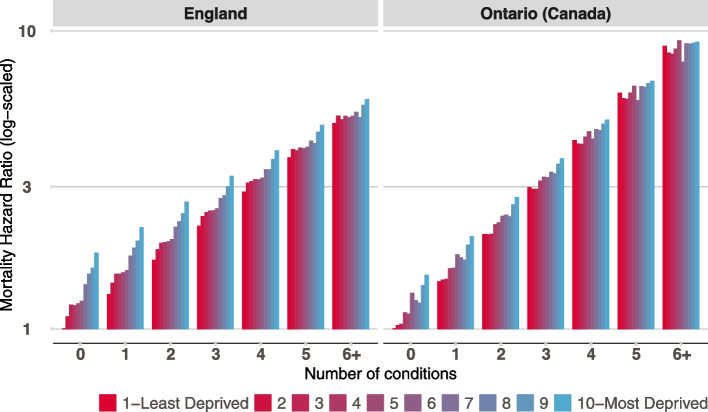
Mortality Hazard Ratio (log scaled) by deprivation and number of conditions for older adults (65 + years) in England and Ontario (Canada). Estimates are shown based a model with sex, deprivation, number of long-term conditions and interaction between deprivation and number of conditions. Mortality hazard ratio of greater than 1 means a higher likelihood of dying during follow up compared to a person from the least deprived areas (decile 1) and no conditions

To test whether the association between number of long-term conditions and mortality differed by the level of deprivation, interactions between number of conditions and deprivation were added in the age-stratified models. Adding the interactions statistically improved the model fit for working age adults in Ontario and older adults living in England and Ontario (Supplementary Table 9 and 10 for full regression coefficients). Adults with more long-term conditions had a higher mortality rate and those living in deprived areas also had a higher mortality rate but for adults living in the most deprived areas, the relative difference in mortality with more vs no conditions was smaller compared to those living in least deprived areas. This means that having more conditions attenuates the deprivation gradient in mortality. This was true for working age adults in Ontario (Fig. 2) and older adults (Fig. 3) in England and Ontario.

The pattern of results was similar in the sensitivity analyses (Supplementary Table 11–12).

## Discussion

### Contribution of our study

Our analysis confirms that having multiple long-term conditions is associated with higher mortality rate. This association was stronger in working age compared with older adults. The analysis also highlights the contribution of multiple long-term conditions to socioeconomic inequalities in the survival of working age and older adults. Number of conditions and socioeconomic deprivation both contribute to higher mortality risk, but number of conditions moderates the socioeconomic gradient in mortality: a shallower gradient was seen for persons with more long-term conditions.

### Comparison with previous research

Our findings based on large population-based samples from two jurisdictions (England and Ontario) add to the evidence that having multiple long-term conditions is strongly associated with a higher mortality rate [13–15] and that the influence of more conditions on mortality is stronger in working age than in older adults [13]. Some previous studies have focused on the interaction of socioeconomic deprivation and number of conditions and our findings align with those that showed increasing number of conditions attenuates socioeconomic inequalities in mortality [27, 29, 32]. We have shown that this is consistent for older adults in England and both working age and older adults in Ontario. Our findings are also similar to a study set in Ontario which found little evidence for an interaction between multiple long-term conditions and socioeconomic factors but a trend towards a stronger association between multiple conditions and mortality amongst individuals living in the highest income areas [31].

Others have found that the influence of multiple long-term conditions on mortality remained consistent across levels of socioeconomic deprivation [13, 28] or shown shorter survival in people with multiple long-term conditions living in the most deprived areas [30]. Our findings contradict these and there are various possible explanations for the differences.

The two studies finding no interaction between multiple long-term conditions and socioeconomic factors [13, 28] were conducted in the UK used UK Biobank data and Whitehall II cohort. These are high quality studies but participants are mainly of white British ethnicity from less deprived groups. The occupational cohort tends to be healthier compared to the general UK population so the findings of those studies may reflect lower socioeconomic diversity with lower condition prevalence rates [13, 28]. So, estimates on outcomes particularly in higher counts of conditions are likely to be conservative [15].

One study conducted in England found shorter survival in men with two or more long-term conditions living in the most compared with the least deprived areas [30]. The authors proposed, though did not test, that sex differences in survival after the onset of multiple long-term conditions could be due to differences in disease combinations. In fact, studies conducted with similar cohorts in Ontario found that disease combinations somewhat varied between men and women within age groups [7].

### Possible mechanisms

Health service factors may contribute to poorer survival of people with multiple long-term conditions. People managing multiple long-term conditions tend to require access to multiple health and social care services, however, they report poorer access to primary care, poorer communication with primary care staff [49] and higher perceived unmet need in primary care and local services [50, 51]. Despite higher health care use such as more frequent primary care and ambulatory care visits[19], people managing multiple long-term conditions experience fragmented care [52] and poorer outcomes such as higher emergency admissions [19], suggesting their needs are not fully met by the current health care system. Also having multiple long-term conditions, particularly a combination of physical and mental health conditions, is associated with lower health literacy [53] suggesting possible difficulties in navigating the complex health system. Interventions that promote self-management with the aim to increase personalised and self-care (e.g., supporting medical adherence or condition-specific education) on their own and in combination with interventions that promote a collaborative process with better communication between patients and clinicians across different care settings (such as case management, multidisciplinary teams and discharge management) have been shown to reduce care fragmentation and improve outcomes for people managing long-term conditions [54].

Some previous studies might lead us to hypothesise that socioeconomic deprivation would exacerbate poor outcomes for individuals with multiple long-term conditions. Individuals from areas of high deprivation have a faster acquisition of additional long-term conditions [55] and are more likely to have complex multimorbidity (three or more conditions affecting three or more different body systems) [2], a combination of physical and mental conditions [56] or frailty [57] that may make it harder to navigate day to day living and care. Qualitative evidence highlights how living with multiple long-term conditions in deprived areas often requires people to manage physical, mental and social problems and challenges their ability to preserve autonomy [58, 59]. For example, having a manual job that is physically exhausting, an unfavourable living situation that make it difficult to access and engage with the necessary support, not being able to speak English that makes it challenging to navigate healthcare and to get a clear understanding of the conditions and treatments they need to manage (i.e., having lower health literacy) often also contribute to the difficulty in adopting healthy behaviours [60]. This suggests that the needs of people with multiple long-term conditions particularly in deprived areas go beyond the management of physical and mental symptoms and therefore require different types of care and self-management techniques. This is echoed by the experience of GPs working in areas of high deprivation where they feel that the current healthcare system and self-management strategies do not meet the patient’s needs and they are not allocated resources to provide the optimal additional support [61]. There are fewer GP practices and GPs per head after adjusting for population need in areas of higher deprivation [62].

However, our results show that the relative difference in mortality between those with no and multiple long-term conditions is smaller in more socioeconomically deprived areas. It is possible that having existing conditions may increase the opportunity to see physicians which may lead to better detection and management of subsequent conditions [63]. It has been suggested that increasing number of conditions may be associated with better quality of care when measured through process or outcome indicators such as all-cause mortality and worse quality of care when measured through patient reported information such as quality of life measures, so, we may not have fully captured the added burden of increasing multiple long-term conditions in deprived areas [64].

On the other hand, the shallower gradient in deprivation for increasing number of conditions may be an expression of “healthy survivor bias”. It could be that people still alive and eligible for inclusion in our study are different from those who have already died with multiple long-term conditions. For example, they may be more likely to present with an accumulation of less lethal conditions such as hypertension or arthritis compared with their counterparts that died before the study start [16, 32, 65–68]. Our study may therefore be underestimating the mortality risk of multiple long-term conditions especially in deprived areas where people acquire these at younger ages [8, 9]. Furthermore, our study assumes that increasing number of conditions represents worsening disease trajectories and clinical complexity whereas the impact on mortality may be smaller for certain conditions [19]. Alternative study designs and longer follow-up are needed to assess worsening disease trajectories including accumulation, severity of conditions that may differ by deprivation [19, 30, 32, 55, 69]. Complexity of care may be mediated by the trajectory and combination of conditions and the degree to which management and treatment of conditions interact with one another [70–72]. Consequently, number of conditions may not be reflective of the complexity of care needed [58, 59]. Currently there is no consensus on the best method to identify patterns or clusters of conditions and there is a lack of research on how clusters and condition trajectories may influence complexity of management [4, 72, 73] Future studies should explore how severity, complexity of clusters of conditions and the accumulation of new conditions may influence the survival of people with multiple long-term conditions particularly in areas of deprivation.

### Strengths and limitations

A strength of our study is the sample sizes of over half a million people in England and in Ontario with a long follow up period. The replication in two jurisdictions with great similarity in the findings is an indication of the robustness of the analysis. Currently, there is no single accepted definition or measurement of multiple long-term conditions [74] and this has previously made it difficult to compare findings [75] but our study captured the same conditions and used the same analytical protocol. Emerging work on establishing a consensus is underway [76] and likely to be cited as the standard for definition and description which will make comparability and reproducibility easier [77].

The use of population-based electronic health records means that we reduced our selection bias, on the other hand we cannot assume accuracy and standardisation of coding in the database [78, 79]. Additionally, routine health records do not include high quality information on severity of conditions, frailty or measures of material, psychosocial or behavioural factors which may contribute to higher mortality of people with multiple long-term conditions. Similarly, we only had access to area-level deprivation, and this is a marker of contextual risk and may not be representative of individual social determinants of health and day to day living. This is a common limitation when using routine health records. Even so, individual and area-level measures of socioeconomic position have shown similar relationship with morbidity [5]. In addition, exploring the rate of development of new conditions during follow up was beyond the scope of this study. Our study period excludes the start of the COVID-19 pandemic. The COVID-19 pandemic disproportionately affected those in the most deprived areas [80] and it also disrupted usual care with an overall reduction in consultations or in appointments [81] and the halt of services designed to monitor new conditions and address inequalities such as the NHS health check [39]. It is possible that our study underestimates the impact of multiple long-term conditions on inequalities in survival in the context of the pandemic.

## Conclusion

Long-term conditions are increasing in prevalence particularly among working age adults in deprived areas in England and Ontario [2, 7]. Having multiple long-term conditions and living in deprived areas is associated with a higher mortality rate in working age and older adults in England and Ontario. Both jurisdictions need to pay greater attention to working age adults with multiple long-term conditions. Currently the health care system has a fragmented approach in the management of multiple long-term conditions which results in high and complex unmet needs, particularly in areas of high deprivation. Evidence suggests that improving the availability, access and relationships with local health and social care services and improving coordination between different services is necessary to help people manage multiple long-term conditions and prevent the development of new conditions particularly in areas of deprivation [60]. There is also a need for health care professionals to better understand the groups experiencing inequalities particularly in their local area so they can encourage individuals to take control of their own health and manage their health conditions, for example, giving targeted recommendations and encouragement on self-management and care based on their social and cultural context [60]. Further work should explore how we can better support patients and clinicians in preventing the development and improving the management of multiple-long term conditions.

## Supplementary Information

**Additional file 1: Supplementary Table 1.** Health administrative data sources used (Ontario). **Supplementary Table 2.** Description of the 2015 Index of Multiple Deprivation (IMD) and 2016 ON-MARG material deprivation index (ON). **Supplementary Table 3.** Time criteria for long-term conditions counted in the current study (including those counted in the sensitivity analysis) in England. **Supplementary Table 4:** Case ascertainment algorithms for long-term conditions, applied to Ontario (Canada) health administrative data sources. **Supplementary Table 5.** Demographic characteristics of people with missing data that were excluded from the analytical sample by jurisdiction. **Supplementary Table 6.** Descriptive table of those censored in England and Ontario (Canada). **Supplementary Table 7.** Prevalence of long-term conditions by jurisdiction. **Supplementary Table 8.** Cox regression estimates for model 1 and 2 in England and Ontario (Canada). **Supplementary Table 9.** Cox regression estimates from model 3 and 4 stratified for working age adults (18–64 years) in England and Ontario (Canada). **Supplementary Table 10.** Cox regression estimates from model 3 and 4 stratified for older adults (65 + years) in England and Ontario (Canada). **Supplementary Table 11.** Cox Regression Estimates from the sensitivity analyses (to include the list of 26 conditions) of model 3 and 4 for working age adults (18–64 years) in England and Ontario (Canada). **Supplementary Table 12.** Cox Regression Estimates from the sensitivity analyses (to include the list of 26 conditions) of model 3 and 4 for older adults (65 + years) in England and Ontario (Canada).

**Additional file 2: Supplementary Figure 1**. Linear association between number of long-term conditions and mortality for England and Ontario (Canada). **Supplementary Figure 2.** Distribution of number of conditions by age group and jurisdiction.

## Data Availability

The data that support the findings of this study are available from the Clinical Practice Research Datalink (CPRD) in England or at ICES in Ontario, but restrictions apply to the availability of these data, which were used under licence for the current study, and so are not publicly available. Data may however be available on application directly to the CPRD or ICES. The analytical code can be found on GitHub: https://github.com/HFAnalyticsLab/MLTCs_deprivation_and_mortality.

## Abbreviations

HES: Hospital Episode Statistics
IMD: Index of Multiple Deprivation
ON-MARG indices: 2016 Material deprivation index in Ontario
CPRD: Clinical Practice Research Datalink
