# Supplementary material for: Socioeconomic gradient in mortality of working age and older adults with multiple long-term conditions in England and Ontario, Canada
Source: BMC Public Health. 2023 Mar 11;23:472. doi: 10.1186/s12889-023-15370-y (PMC10008074; doi:10.1186/s12889-023-15370-y)
Supplement: Supplementary file 1 — Additional file 1: Supplementary Table 1. Health administrative data sources used (Ontario). Supplementary Table 2. Description of the 2015 Index of Multiple Deprivation (IMD) and 2016 ON-MARG material deprivation index (ON). Supplementary Table 3. Time criteria for long-term conditions counted in the current study (including those counted in the sensitivity analysis) in England. Supplementary Table 4: Case ascertainment algorithms for long-term conditions, applied to Ontario (Canada) health administrative data sources. Supplementary Table 5. Demographic characteristics of people with missing data that were excluded from the analytical sample by jurisdiction. Supplementary Table 6. Descriptive table of those censored in England and Ontario (Canada). Supplementary Table 7. Prevalence of long-term conditions by jurisdiction. Supplementary Table 8. Cox regression estimates for model 1 and 2 in England and Ontario (Canada). Supplementary Table 9. Cox regression estimates from model 3 and 4 stratified for working age adults (18–64 years) in England and Ontario (Canada). Supplementary Table 10. Cox regression estimates from model 3 and 4 stratified for older adults (65 + years) in England and Ontario (Canada). Supplementary Table 11. Cox Regression Estimates from the sensitivity analyses (to include the list of 26 conditions) of model 3 and 4 for working age adults (18–64 years) in England and Ontario (Canada). Supplementary Table 12. Cox Regression Estimates from the sensitivity analyses (to include the list of 26 conditions) of model 3 and 4 for older adults (65 + years) in England and Ontario (Canada). [file 12889_2023_15370_MOESM1_ESM.docx]

**Additional file 1 legend:**

**Supplementary Table 1:** Health administrative data sources used (Ontario).

**Supplementary Table 2.** Description of the 2015 Index of Multiple Deprivation (IMD) and 2016 ON-MARG material deprivation index (ON).

**Supplementary Table 3.** Time criteria for long-term conditions counted in the current study (including those counted in the sensitivity analysis) in England.

**Supplementary Table 4:** Case ascertainment algorithms for long-term conditions, applied to Ontario (Canada) health administrative data sources.

**Supplementary Table 5.** Demographic characteristics of people with missing data that were excluded from the analytical sample by jurisdiction.

**Supplementary Table 6.** Descriptive table of those censored in England and Ontario (Canada).

**Supplementary Table 7.** Prevalence of long-term conditions by jurisdiction.

**Supplementary Table 8.** Cox regression estimates for model 1 and 2 in England and Ontario (Canada).

**Supplementary Table 9.** Cox regression estimates from model 3 and 4 stratified for working age adults (18-64 years) in England and Ontario (Canada).

**Supplementary Table 10.** Cox regression estimates from model 3 and 4 stratified for older adults (65+ years) in England and Ontario (Canada).

**Supplementary Table 11.** Cox Regression Estimates from the sensitivity analyses (to include the list of 26 conditions) of model 3 and 4 for working age adults (18-64 years) in England and Ontario (Canada).

**Supplementary Table 12.** Cox Regression Estimates from the sensitivity analyses (to include the list of 26 conditions) of model 3 and 4 for older adults (65+ years) in England and Ontario (Canada).

**Supplementary Table 1:** Health administrative data sources used (Ontario).

| **Data Source** | **Abbreviation** | **Description** |
| --- | --- | --- |
| Registered Persons Database | RPDB | A population-based registry that contains demographic information for all residents of Ontario who have registered for health insurance. |
| Postal Code Conversion File | PCCF | Converts postal code data to other standard geographical areas |
| Ontario Marginalization database | ONMARG | Geographical (census) based indices developed to quantify the degree of marginalization, measured by 4 distinct dimensions including material deprivation, across the province of Ontario |
| Ontario Health Insurance Program | OHIP | Claims for all physician services provided to Ontario residents paid for by the MOHLTC |
| Discharge Abstract Database | DAD | Administrative and clinical information on all admissions/discharges from acute care facilities in Ontario |
| Same Day Surgery | SDS | Administrative and clinical information on all same-day surgeries or procedure stays from surgery institutions in Ontario |
| Ontario Drug Benefits database | ODB | Claims for all prescription drugs received under the Ontario Drug Benefit program (primarily for persons aged 65 years and older) |
| National Ambulatory Care Reporting System | NACRS | Visits for all hospital- and community-based ambulatory care  services (day surgery, outpatient clinic visits, emergency department visits) provided to Ontario residents |
| Ontario Mental Health Reporting System | OMHRS | Administrative and clinical information on adult designated inpatient mental health stays |
| Asthma | ASTHMA_SPEC | An ICES-derived cohort of persons diagnosed with asthma, using a definition with increased specificity |
| CHF | CHF | An ICES-derived cohort of persons diagnosed with congestive heart failure |
| COPD | COPD_SPECIFIC | An ICES-derived cohort of persons diagnosed with chronic obstructive pulmonary disease, using a definition with increased specificity |
| Dementia | DEMENTIA | An ICES-derived cohort of persons diagnosed with dementia |
| Ontario Diabetes Database | ODD | An ICES-derived cohort of persons diagnosed with diabetes |
| Hypertension | HYPERTENSION | An ICES-derived cohort of persons diagnosed with hypertension |
| Ontario Rheumatoid Arthritis Database | ORAD | An ICES-derived cohort of persons diagnosed with (rheumatoid) arthritis |
| Ontario Cancer Registry | OCR | A provincial database including information on all Ontario residents diagnosed with cancer |

**Supplementary Table 2.** Description of the 2015 Index of Multiple Deprivation (IMD) and 2016 ON-MARG material deprivation index (ON).

| 2015 Index of Multiple Deprivation: Domains (domains produce an overall relative measure of deprivation, domains are weighted) (33) | 2016 ON-MARG material deprivation index: Dimensions (34) |
| --- | --- |
| 1. Education Skills, Training Deprivation- measures the lack of attainment and skills in the local population 2. Living Environment Deprivation- measures the quality of the local environment 3. Income Deprivation- proportion of the population experiencing deprivation relating to low income 4. Employment Deprivation- proportion of the working age population in an area involuntarily excluded from the labour market. 5. Barriers to Housing and Services- measures the physical and financial accessibility of housing and local services 6. Health Deprivation and Disability- measures the risk of premature death and the impairment of quality of life through poor physical or mental health 7. Crime- measures the risk of personal and material victimisation at local level | 1. Education - Proportion of the population without a high school diploma 2. Family Structure - Proportion of families who are lone parent families 3. Income - Proportion of total income from government transfer payments for population age 15y+ 4. Income - Proportion of the population aged 15y+ that are unemployed 5. Income - Proportion of the population considered low income 6. Housing- Proportion of households living in dwellings that are in need of major repair |

**Supplementary Table 3.** Time criteria for long-term conditions counted in the current study (including those counted in the sensitivity analyses) in England.

| **Condition** | **Medcodes (Diagnosis codes)** | **Prodcodes (Treatment codes)** | **Time criteria** |
| --- | --- | --- | --- |
| Alcohol Misuse | x |  | If it ever existed in their history before baseline |
| Arthritis (Rheumatoid) | x |  |  |
| Asthma | x | x | In the last 12 months before baseline |
| Atrial fibrillation | x |  | If it ever existed in their history before baseline |
| Cancer (Any) | x |  | If it ever existed in their history in the last five years before baseline |
| Chronic Obstructive Pulmonary Disease | x |  | If it ever existed in their history before baseline |
| Congestive Heart Failure | x |  | If it ever existed in their history before baseline |
| Dementia | x |  | If it ever existed in their history before baseline |
| Diabetes | x |  | If it ever existed in their history before baseline |
| Epilepsy | x |  | If it ever existed in their history before baseline |
| Ischemic Heart Disease | x |  | If it ever existed in their history before baseline |
| Hypertension | x |  | If it ever existed in their history before baseline |
| Kidney disease | x |  | If it ever existed in their history before baseline |
| Multiple Sclerosis | x |  | If it ever existed in their history before baseline |
| Parkinsons | x |  | If it ever existed in their history before baseline |
| Psoriasis | x |  | If it ever existed in their history before baseline |
| Schizophrenia | x |  | If it ever existed in their history before baseline |
| Stroke (TIA) | x |  | If it ever existed in their history before baseline |
| Thyroid disorders | x |  | If it ever existed in their history before baseline |
| **Other conditions only included in the sensitivity analyses** |  |  |  |
| Anxiety and Depression | x | x | Medcodes in the last 12 months before our baseline or at least 4 prodcodes in the last 12 months before our baseline |
| Blindness | x |  | If it ever existed in their history before baseline |
| Bronchiectasis | x |  | If it ever existed in their history before baseline |
| Diverticulosis | x |  | If it ever existed in their history before baseline |
| Hearing loss | x |  | If it ever existed in their history before baseline |
| Liver Disease | x |  | If it ever existed in their history before baseline |
| Substance misuse | x |  | If it ever existed in their history before baseline |

*only included in the sensitivity analyses

Medcodes and validation can be found here: <https://github.com/annalhead/CPRD_multimorbidity_codelists>

**Supplementary Table 4:** Case ascertainment algorithms for long-term conditions, applied to Ontario (Canada) health administrative data sources.

| **Condition** | **ICD9 / OHIP** | **ICD10** | **ODB** | **Algorithm or Data Used [exclusions]** | **Reference** |
| --- | --- | --- | --- | --- | --- |
| Alcohol Misuse | OHIP: 291, 303  DAD: 291, 303, 305.0 | F10 |  | 2P in 365d window or 1H (ages 20+) | None.  Adapted from Hulme (2020) Mortality among patients with frequent ED use for alcohol-related reasons in ON; CMAJ 192(47): E1522 and Bolton (2020) Health service use among Manitobans with alcohol use disorder: a population-based matched cohort study. CMAJ Open; 8(4): E762 |
| (Rheumatoid) Arthritis | 714 | M05, M06 |  | ORAD | Widdifield J, et al. An administrative data validation study of the accuracy of algorithms for identifying rheumatoid arthritis: the influence of the reference standard on algorithm performance. BMC musculoskeletal disorders. 2014 Jun 23;15(1):216 |
| Asthma | 493 | J45, J46 |  | ASTHMA | Gershon AS, et al. Identifying patients with physician-diagnosed asthma in health administrative databases. Can Respir J 2009;16:183–8 |
| Atrial Fibrillation | DAD/NACRS: 427.31, 427.32  OHIP: 427 | I48 |  | 1H or 1ED or 4P in 365d with claims 30d apart | Tu K, et al. Identifying patients with atrial fibrillation in administrative data. Can J Cardiol 2016; 32(12): 1561-5 |
| (any) Cancer | n/a | n/a |  | OCR  *Diagnosis in last 5 years only* | None. |
| Chronic Obstructive Pulmonary Disease | 491, 492, 496 | J41, J43, J44 |  | COPD_SPECIFIC | Gershon AS, et al. Identifying Individuals with Physician Diagnosed COPD in Health Administrative Databases. Copd 2009;6:388–94 |
| Congestive Heart Failure | 428 | I500, I501, I509 |  | CHF | Schultz SE, et al Identifying cases of congestive heart failure from administrative data: a validation study using primary care patient records. Chronic Diseases and Injuries in Canada 2013;33:160–6. |
| Dementia | 290, 331 (OHIP) / 046.1, 290.0, 290.1, 290.2, 290.3, 290.4, 294, 331.0, 331.1, 331.5, F331.82 (DAD) | F00, F01, F02, F03, G30 | Cholinesterase Inhibitors | DEMENTIA | Jaakkimainen RL, et al. Identification of Physician-Diagnosed Alzheimer's Disease and Related Dementias in Population-Based Administrative Data: A Validation Study Using Family Physicians' Electronic Medical Records. J Alzheimers Dis. IOS Press; 2016 Aug 10;54(1):337–49 |
| Diabetes | 250 | E08 - E13 | Oral anti-glycemics, Insulin, Anti-diabetic agents: Misc | ODD | Hux JE, et al. Diabetes in Ontario: Determination of prevalence and incidence using a validated administrative data algorithm. Diabetes Care 2002;25:512–6 |
| Epilepsy | 345 | G40 |  | 3P in 2yr window with claims 30+ days apart (ages 1-19); OR  3P in 2yr window with claims 30+ days apart or 1H (ages 20+) | CCDSS* |
| (Ischemic) Heart Disease | 410, 411, 412, 413, 414 | I20, I21, I22, I23, I24, I25 |  | 2P in 365d window or 1H (ages 20+) | CCDSS* |
| Hypertension | 401, 402, 403, 404, 405 | I10, I11, I12, I13, I15 |  | HYPER | Tu K, et al. Accuracy of administrative databases in identifying patients with hypertension. Open Med 2007;1:e18–26 |
| (Chronic) Kidney Disease | DAD: 403.0, 403.1, 403.9, 404.0, 404.1, 404.9, 585, 586, 588.8, 588.9, 250.4  OHIP: 403, 585 | E102, E112, E132, E142, I12, I13, N08, N18, N19 |  | 2P in 365d window or 1H (ages 20+) | None. |
| Multiple Sclerosis | 340 | G35 |  | 5P in 2yr window or 1H (ages 20+) | CCDSS* |
| Parkinsons | 332 |  |  | 3P in 2yr window with claims 30+ days apart (ages 20+) | Modified CCDSS* |
| Psoriasis | DAD: 696.1, 696.8  OHIP: 696 | L40.0, L40.1, L40.2, L40.3, L40.4, L40.8, L40.9 |  | 2P in 365d window or 1H (ages 20+) | Eder L, et al. Identifying and characterizing psoriasis and psoriatic arthritis patients in Ontario administrative data: a population-based study from 1991 to 2015. The Journal of Rheumatology 2020; in press. |
| Schizophrenia | 295 | F20, F21, F23, F25 |  | 2P in 365d window with claims 30+ days apart or 1H (ages 10+) | CCDSS* |
| Stroke (excl TIA) | DAD: 325, 362.3x, 430, 431, 432.9, 433.x1, 434 (or 434.x1), 435.x, 436, 437.6  OHIP: 325, 430, 431, 434, 435, 436 | G08, G45.x (exclude G45.4), H34.0, H34.1, I60.x, I61.x, I62.9, I63.x, I64, I67.6 |  | 2P in 365d window or 1H (ages 20+) | CCDSS* |
| Thyroid Disorders | 240-246 | E00-E07 |  | 2P in 365d window or 1H (ages 20+) | None.  Adapted from: Fortin M et al. Self-reported versus health administrative data: implications for assessing  chronic illness burden in populations. A cross-sectional study. CMAJ Open 2017. DOI:10.9778/cmajo.20170029. |
| **Other Conditions only included in sensitivity analyses** |  |  |  |  |  |
| Blindness / Visual Impairment | 369 | H54 |  | 2P in 365d window or 1H (ages 20+) | None.  Adapted from: Brown HK, Ray JG, Chen S, et al. Association of preexisting disability with severe maternal  morbidity or mortality in Ontario, Canada. JAMA Netw Open. 4(2):e2034993. |
| Bronchiectasis | 494 | J47 |  | 2P in 365d window or 1H (ages 20+) | None. |
| Cirrhosis | OHIP: 571  ICD9: 456.1, 571.2, 571.5 | I85.9, I98.2, K70.3, K71.7, K74.6 |  | 2P* in 5yr window with claims 1+ days apart or 1H | Lapointe-Shaw L (2018) Identifying cirrhosis, decompensated cirrhosis and hepatocellular carcinoma in health administrative data: A validation study; 13(8): e0201120. |
| Diverticulosis | 562 | K57 |  | 2P in 365d window or 1H (ages 20+) | None.  Adapted from: Warner E (2007) Fourteen-year study of hospital admissions for diverticular disease in Ontario. Can J Gastenterol; 21(2): 91-99 |
| Hearing Loss / Impairment | 389 | H90, H91.3, H91.8, H91.9 |  | 2P in 365d window or 1H (ages 20+) | None.  Adapted from: Brown HK, Ray JG, Chen S, et al. Association of preexisting disability with severe maternal  morbidity or mortality in Ontario, Canada. JAMA Netw Open. 4(2):e2034993. |
| Mood and Anxiety Disorders | 296, 300, 311 | F30-F42, F44-F48, F68 |  | 1P or 1H in last 365d  *Diagnosis in last 12 months only* | CCDSS* |
| Substance Misuse | 304 | F112, F122, F132, F142, F152, F162, F192 |  | 2P in 365d window or 1H (ages 20+) | None. |

*full details of conditions captured in the Canadian Chronic Disease Surveillance System (CCDSS) can be [downloaded here](https://health-infobase.canada.ca/ccdss/publication/CCDSS_Case_Definitions_DataCubes_v2018_en.xlsx) [accessed August 01, 2021].

P = physician billings, use OHIP data (excluding codes originating from medical laboratories)

H = hospital admission, use DAD/SDS data (including all primary and secondary diagnoses in the records)

Rx = prescription drug claim, use OBD data

ED = emergency department visit, use NACRS data

MHH = mental health hospitalization, use OMHRS_DAD data

Unless otherwise specified, a minimum age of 20+ is used at time of diagnosis.

Where no validated algorithm exists, we adopted a 1H or 2P in 365 day period approach, which is the general standard in the CCDSS

**Supplementary Table 5.** Demographic characteristics of people with missing data that were excluded from the analytical sample by jurisdiction.

|  | England | | Ontario (Canada) | |
| --- | --- | --- | --- | --- |
|  | Analytical Sample | Excluded due to missing Data | Analytical sample | Excluded due to missing Data |
| n | 599,487 | 513 | 594,526 | 5,474 |
| Baseline age N (%) |  |  |  |  |
| 18-64 years | 462,761 (77.2%) | 413 (80.5%) | 474,824 (79.9%) | 4,468 (81.6%) |
| 65+ years | 136,726.0 (22.8%) | 100 (19.5%) | 119,702 (20.1%) | 1,006 (18.4%) |
| Women N (%) | 300,098 (50.1) | 249 (48.5) | 305,309 (51.4%) | 2,629 (48.0%) |
| Baseline number of long-term conditions mean (sd) | 0.70 (1.11) | 0.67 (1.14) | 0.90 (1.31) | 0.98 (1.43) |

**Supplementary Table 6.** Descriptive table of those censored in England and Ontario (Canada).

|  | **England** | **Ontario (Canada)** |
| --- | --- | --- |
| N | 139,090 | 9,312 |
| Baseline age N (%) |  |  |
| Working age adults (18-64 years) | 122,820 (88.3%) | 8,449 (90.7%) |
| Older adults (65+ years) | 16,270 (11.7%) | 863 (9.3%) |
| Women N (%) | 71,063 (51.1%) | 4,421 (47.5%) |
| Deprivation Decile |  |  |
| 1- Least Deprived | 12,909 (9.3%) | 1,207 (13.0%) |
| 2 | 13,262 (9.5%) | 1,048 (11.3%) |
| 3 | 14,640 (10.5%) | 979 (10.5%) |
| 4 | 13,831 (9.9%) | 936 (10.1%) |
| 5 | 13,094 (9.4%) | 833 (8.9%) |
| 6 | 13,949 (10.0%) | 841 (9.0%) |
| 7 | 15,838 (11.4%) | 874 (9.4%) |
| 8 | 14,996 (10.8%) | 863 (9.3%) |
| 9 | 14,677 (10.6%) | 861 (9.2%) |
| 10- Most Deprived | 11,894 (8.6%) | 870 (9.3%) |
| Baseline number of long-term conditions mean (sd) | 0.44 (0.85) | 0.46 (0.90) |

**Supplementary Table 7.** Prevalence of long-term conditions by jurisdiction.

| **Condition** | **England (N, %)** | **Ontario (Canada) (N, %)** |
| --- | --- | --- |
| Alcohol | 4,536 (0.8%) | 12,582 (2.1%) |
| Arthritis (Rheumatoid) | 4,641 (0.8%) | 6,398 (1.1%) |
| Asthma | 68,384 (11%) | 57,917 (9.7%) |
| Atrial fibrillation | 14,859 (2.5%) | 16,878 (2.8%) |
| Cancer (Any) | 12,382 (2.1%) | 12,542 (2.1%) |
| Chronic Obstructive Pulmonary Disease | 14,049 (2.3%) | 16,016 (2.7%) |
| Congestive Heart Failure | 7,315 (1.2%) | 13,570 (2.3%) |
| Dementia | 4,763 (0.8%) | 9,856 (1.7%) |
| Diabetes | 41,047 (6.8%) | 68,636 (11.5%) |
| Epilepsy | 9,129 (1.5%) | 6,424 (1.1%) |
| Ischemic Heart Disease | 28,587 (4.8%) | 54,964 (9.2%) |
| Hypertension | 110,131 (18%) | 159,837 (26.9%) |
| Kidney disease | 28,353 (4.7%) | 12,572 (2.1%) |
| Multiple Sclerosis | 1,527 (0.3%) | 1,546 (0.3%) |
| Parkinsons | 1,448 (0.2%) | 1,609 (0.3%) |
| Psoriasis | 21,311 (3.6%) | 11,778 (2.0%) |
| Schizophrenia | 2,157 (0.4%) | 6,788 (1.1%) |
| Stroke (TIA) | 14,189 (2.4%) | 18,095 (3.0%) |
| Thyroid disorders | 30,370 (5.1%) | 48,850 (8.2%) |
| **Other conditions only included in the sensitivity analyses** |  |  |
| Anxiety and Depression | 54,629 (9.1%) | 73,775 (12.4%) |
| Blindness | 5,301 (0.9%) | 1,594 (0.3%) |
| Bronchiectasis | 2,644 (0.4%) | 1,104 (0.2%) |
| Diverticulosis | 18,862 (3.1%) | 34,346 (5.8%) |
| Hearing loss | 45,371 (7.6%) | 21,412 (3.6%) |
| Liver Disease | 4,056 (0.7%) | 3,279 (0.6%) |
| Substance misuse | 1,908 (0.3%) | 16,428 (2.8%) |

**Supplementary Table 8.** Cox regression estimates for model 1 and 2 in England and Ontario (Canada).

|  |  | England | | | | Ontario (Canada) | | | |
| --- | --- | --- | --- | --- | --- | --- | --- | --- | --- |
|  |  | Model 1 | | Model 2 | | Model 1 | | Model 2 | |
| Covariate | Reference group | Regression Estimate | Standard error | Regression Estimate | Standard error | Regression Estimate | Standard error | Regression Estimate | Standard error |
| Women | Men | -0.27^**^ | 0.01 | -0.27^**^ | 0.01 | -0.35^**^ | 0.012 | -0.35^**^ | 0.01 |
| Age |  | 0.11^**^ | 0.00 | 0.12^**^ | 0.002 | 0.10^**^ | 0.00 | 0.11^**^ | 0.002 |
| Deprivation | 1- Least Deprived |  |  |  |  |  |  |  |  |
| 2 |  | 0.13^**^ | 0.02 | 0.28^**^ | 0.07 | 0.001 | 0.028 | 0.31 | 0.17 |
| 3 |  | 0.18^**^ | 0.02 | 0.34^**^ | 0.07 | 0.003 | 0.029 | 0.37^*^ | 0.17 |
| 4 |  | 0.20^**^ | 0.02 | 0.41^**^ | 0.07 | 0.11^**^ | 0.028 | 0.66^**^ | 0.17 |
| 5 |  | 0.21^**^ | 0.02 | 0.44^**^ | 0.07 | 0.13^**^ | 0.028 | 0.56^**^ | 0.17 |
| 6 |  | 0.25^**^ | 0.02 | 0.68^**^ | 0.06 | 0.15^**^ | 0.028 | 0.89^**^ | 0.16 |
| 7 |  | 0.35^**^ | 0.02 | 0.84^**^ | 0.06 | 0.21^**^ | 0.028 | 1.14^**^ | 0.16 |
| 8 |  | 0.43^**^ | 0.03 | 1.05^**^ | 0.06 | 0.23^**^ | 0.027 | 1.54^**^ | 0.16 |
| 9 |  | 0.52^**^ | 0.02 | 1.16^**^ | 0.06 | 0.34^**^ | 0.00 | 1.90^**^ | 0.15 |
| 10- Most Deprived | | 0.66^**^ | 0.02 | 1.42^**^ | 0.06 | 0.44^**^ | 0.00 | 2.25^**^ | 0.15 |
| Age x Deprivation interaction | 1-Least Deprived |  |  |  |  |  |  |  |  |
| 2 |  |  |  | -0.01^*^ | 0.002 |  |  | -0.004 | 0.002 |
| 3 |  |  |  | -0.01^*^ | 0.002 |  |  | -0.01^*^ | 0.002 |
| 4 |  |  |  | -0.01^*^ | 0.002 |  |  | -0.01^*^ | 0.002 |
| 5 |  |  |  | -0.01^**^ | 0.002 |  |  | -0.01^*^ | 0.002 |
| 6 |  |  |  | -0.02^**^ | 0.002 |  |  | -0.01^**^ | 0.002 |
| 7 |  |  |  | -0.02^**^ | 0.002 |  |  | -0.01^**^ | 0.002 |
| 8 |  |  |  | -0.02^**^ | 0.002 |  |  | -0.012^**^ | 0.002 |
| 9 |  |  |  | -0.02^**^ | 0.002 |  |  | -0.02^**^ | 0.002 |
| 10-Most Deprived |  |  |  | -0.03^**^ | 0.002 |  |  | -0.02^**^ | 0.002 |
| Likelihood ratio test between Model 1 and Model 2 | | *X*^2^(9) = 420.36, p<0.001 | | | | *X*^2^(9) = 304.45, p<0.001 | | | |

^*^denotes significance at p<0.05, ^**^denotes significance at p<0.001

**Supplementary Table 9.** Cox regression estimates from model 3 and 4 stratified for working age adults (18-64 years) in England and Ontario (Canada).

|  |  | England | | | | Ontario (Canada) | | | |
| --- | --- | --- | --- | --- | --- | --- | --- | --- | --- |
|  |  | Model 3 | | Model 4 | | Model 3 | | Model 4 | |
| Covariate | Reference | Regression Estimate | Standard error | Regression Estimate | Standard error | Regression Estimate | Standard error | Regression Estimate | Standard error |
| Age |  | 0.07^**^ | 0.00 | 0.07^**^ | 0.002 | 0.05** | 0.00 | 0.05^**^ | 0.001 |
| Women | Men | -0.37^**^ | 0.03 | -0.36^**^ | 0.03 | -0.49** | 0.03 | -0.49^**^ | 0.026 |
| Deprivation | 1-Least Deprived |  |  |  |  |  |  |  |  |
| 2 |  | 0.24^**^ | 0.07 | 0.35^**^ | 0.09 | -0.01 | 0.07 | 0.07 | 0.091 |
| 3 |  | 0.23^**^ | 0.07 | 0.35^**^ | 0.09 | -0.01 | 0.07 | 0.04 | 0.093 |
| 4 |  | 0.35^**^ | 0.07 | 0.48^**^ | 0.09 | 0.13^*^ | 0.06 | 0.21^*^ | 0.09 |
| 5 |  | 0.35^**^ | 0.07 | 0.38^**^ | 0.09 | 0.16^*^ | 0.06 | 0.21^*^ | 0.092 |
| 6 |  | 0.39^**^ | 0.07 | 0.47^**^ | 0.09 | 0.18^*^ | 0.06 | 0.30^*^ | 0.09 |
| 7 |  | 0.51^**^ | 0.07 | 0.61^**^ | 0.09 | 0.25^**^ | 0.06 | 0.32^**^ | 0.09 |
| 8 |  | 0.66^**^ | 0.07 | 0.81^**^ | 0.09 | 0.36^**^ | 0.06 | 0.46^**^ | 0.087 |
| 9 |  | 0.73^**^ | 0.06 | 0.86^**^ | 0.09 | 0.47^**^ | 0.06 | 0.66^**^ | 0.085 |
| 10 |  | 0.97^**^ | 0.06 | 1.10^**^ | 0.08 | 0.61^**^ | 0.06 | 0.80^**^ | 0.082 |
| Baseline number of conditions | No conditions | 0.47^**^ | 0.01 | 0.58^**^ | 0.04 | 0.53^**^ | 0.01 | 0.60^**^ | 0.03 |
| Deprivation and baseline number of conditions interaction | 1-Least Deprived and no conditions |  |  |  |  |  |  |  |  |
| 2 and per 1 condition increase | |  |  | -0.11 | 0.06 |  |  | -0.05 | 0.041 |
| 3 and per 1 condition increase | |  |  | -0.12^*^ | 0.06 |  |  | -0.04 | 0.041 |
| 4 and per 1 condition increase | |  |  | -0.13^*^ | 0.06 |  |  | -0.06 | 0.04 |
| 5 and per 1 condition increase | |  |  | -0.04 | 0.06 |  |  | -0.04 | 0.04 |
| 6 and per 1 condition increase | |  |  | -0.09 | 0.05 |  |  | -0.08 | 0.039 |
| 7 and per 1 condition increase | |  |  | -0.1 | 0.05 |  |  | -0.05 | 0.038 |
| 8 and per 1 condition increase | |  |  | -0.14^*^ | 0.05 |  |  | -0.07 | 0.037 |
| 9 and per 1 condition increase | |  |  | -0.12^*^ | 0.05 |  |  | -0.12^**^ | 0.036 |
| 10-Most Deprived and per 1 condition increase | |  |  | -0.13^*^ | 0.05 |  |  | -0.11^**^ | 0.035 |
| Likelihood ratio test between Model 3 and Model 4 | | *X*^2^(9) = 12.07, p=0.21 | | | | *X*^2^(9) = 19.46, p<0.05 | | | |

^*^denotes significance at p<0.05, ^**^ denotes significance at p<0.001

**Supplementary Table 10.** Cox regression estimates from model 3 and 4 stratified for older adults (65+ years) in England and Ontario (Canada).

|  |  | England | | | | Ontario (Canada) | | | |
| --- | --- | --- | --- | --- | --- | --- | --- | --- | --- |
|  |  | Model 3 | | Model 4 | | Model 3 | | Model 4 | |
| Covariate | Reference | Regression Estimate | Standard error | Regression Estimate | Standard error | Regression Estimate | Standard error | Regression Estimate | Standard error |
| Age |  | 0.11^**^ | 0.001 | 0.11^**^ | 0.001 | 0.09^**^ | 0.001 | 0.094^**^ | 0.001 |
| Women | Men | -0.25^**^ | 0.01 | -0.25^**^ | 0.01 | -0.27^**^ | 0.01 | -0.268^**^ | 0.014 |
| Deprivation | 1-Least Deprived |  |  |  |  |  |  |  |  |
| 2 |  | 0.08^*^ | 0.03 | 0.09^*^ | 0.05 | -0.02 | 0.03 | 0.025 | 0.068 |
| 3 |  | 0.12^**^ | 0.03 | 0.18^**^ | 0.05 | -0.02 | 0.03 | 0.034 | 0.069 |
| 4 |  | 0.13^**^ | 0.03 | 0.18^**^ | 0.05 | 0.05 | 0.03 | 0.123 | 0.067 |
| 5 |  | 0.14^**^ | 0.03 | 0.19^**^ | 0.05 | 0.07^*^ | 0.03 | 0.115 | 0.069 |
| 6 |  | 0.15^**^ | 0.03 | 0.21^**^ | 0.05 | 0.05 | 0.03 | 0.274^**^ | 0.067 |
| 7 |  | 0.24^**^ | 0.03 | 0.34^**^ | 0.05 | 0.11^**^ | 0.03 | 0.217^*^ | 0.067 |
| 8 |  | 0.27^**^ | 0.03 | 0.42^**^ | 0.05 | 0.10^*^ | 0.03 | 0.197^*^ | 0.067 |
| 9 |  | 0.33^**^ | 0.03 | 0.47^**^ | 0.05 | 0.16^**^ | 0.03 | 0.335^**^ | 0.066 |
| 10 |  | 0.41^**^ | 0.03 | 0.59^**^ | 0.05 | 0.19^**^ | 0.03 | 0.414^**^ | 0.067 |
| Baseline number of conditions | No conditions | 0.23^**^ | 0.004 | 0.26^**^ | 0.01 | 0.33^**^ | 0.004 | 0.364^**^ | 0.013 |
| Deprivation and baseline number of conditions interaction | 1-Least Deprived and no conditions |  |  |  |  |  |  |  |  |
| 2 and per 1 condition increase | |  |  | -0.01 | 0.02 |  |  | -0.01 | 0.018 |
| 3 and per 1 condition increase | |  |  | -0.03 | 0.02 |  |  | -0.02 | 0.018 |
| 4 and per 1 condition increase | |  |  | -0.02 | 0.02 |  |  | -0.02 | 0.018 |
| 5 and per 1 condition increase | |  |  | -0.02 | 0.02 |  |  | -0.01 | 0.018 |
| 6 and per 1 condition increase | |  |  | -0.03 | 0.02 |  |  | -0.07^**^ | 0.018 |
| 7 and per 1 condition increase | |  |  | -0.04^*^ | 0.02 |  |  | -0.03 | 0.018 |
| 8 and per 1 condition increase | |  |  | -0.06^**^ | 0.02 |  |  | -0.03 | 0.018 |
| 9 and per 1 condition increase | |  |  | -0.05^*^ | 0.02 |  |  | -0.05^*^ | 0.017 |
| 10-Most Deprived and per 1 condition increase | |  |  | -0.07^**^ | 0.02 |  |  | -0.06^**^ | 0.017 |
| Likelihood ratio test | | *X*^2^(9) = 33.66, p<0.001 | | | | *X*^2^(9) = 31.06, p<0.001 | | | |

^*^denotes significance at p<0.05, ^**^ denotes significance at p<0.001

**Supplementary Table 11.** Cox Regression Estimates from the sensitivity analyses (to include the list of 26 conditions) of model 3 and 4 for working age adults (18-64 years) in England and Ontario (Canada).

|  |  | Working age adults (18-64 years) | | | | | | | |
| --- | --- | --- | --- | --- | --- | --- | --- | --- | --- |
|  |  | England | | | | Ontario (Canada) | | | |
| Covariate | Reference | Model 3 | Standard error | Model 4 | Standard error | Model 3 | Standard error | Model 4 | Standard error |
| Age |  | 0.07^**^ | 0.002 | 0.07^**^ | 0.002 | 0.05^**^ | 0.001 | 0.05^**^ | 0.001 |
| Women | Men | -0.40^**^ | 0.03 | -0.40^**^ | 0.03 | -0.51^**^ | 0.03 | -0.51^**^ | 0.03 |
| Deprivation | 1 |  |  |  |  |  |  |  |  |
| 2 |  | 0.23^**^ | 0.07 | 0.36^*^ | 0.10 | -0.01 | 0.07 | 0.13 | 0.10 |
| 3 |  | 0.22^*^ | 0.07 | 0.34^**^ | 0.10 | -0.01 | 0.07 | 0.12 | 0.10 |
| 4 |  | 0.34^**^ | 0.07 | 0.48^**^ | 0.10 | 0.12 | 0.06 | 0.26^*^ | 0.10 |
| 5 |  | 0.33^**^ | 0.07 | 0.37^**^ | 0.10 | 0.15^*^ | 0.06 | 0.28^*^ | 0.10 |
| 6 |  | 0.37^**^ | 0.07 | 0.47^**^ | 0.10 | 0.18^*^ | 0.06 | 0.39^**^ | 0.10 |
| 7 |  | 0.48^**^ | 0.07 | 0.61^**^ | 0.09 | 0.25^**^ | 0.06 | 0.35^**^ | 0.10 |
| 8 |  | 0.63^**^ | 0.07 | 0.79^**^ | 0.09 | 0.35^**^ | 0.06 | 0.49^**^ | 0.10 |
| 9 |  | 0.69^**^ | 0.06 | 0.83^**^ | 0.09 | 0.45^**^ | 0.06 | 0.68^**^ | 0.09 |
| 10 |  | 0.91^**^ | 0.06 | 1.03^**^ | 0.09 | 0.58^**^ | 0.06 | 0.81^**^ | 0.09 |
| Baseline number of conditions | No conditions | 0.45** | 0.01 | 0.54^**^ | 0.04 | 0.50^**^ | 0.01 | 0.58^**^ | 0.03 |
| Deprivation and baseline number of conditions interaction | 1-Least Deprived and no conditions |  |  |  |  |  |  |  |  |
| 2 and per 1 condition increase | |  |  | -0.10^*^ | 0.05 |  |  | -0.07 | 0.04 |
| 3 and per 1 condition increase | |  |  | -0.09 | 0.05 |  |  | -0.07 | 0.04 |
| 4 and per 1 condition increase | |  |  | -0.11^*^ | 0.05 |  |  | -0.07 | 0.04 |
| 5 and per 1 condition increase | |  |  | -0.04 | 0.05 |  |  | -0.07 | 0.04 |
| 6 and per 1 condition increase | |  |  | -0.08 | 0.05 |  |  | -0.11^*^ | 0.04 |
| 7 and per 1 condition increase | |  |  | -0.10^*^ | 0.05 |  |  | -0.06 | 0.03 |
| 8 and per 1 condition increase | |  |  | -0.11^*^ | 0.04 |  |  | -0.07^*^ | 0.03 |
| 9 and per 1 condition increase | |  |  | -0.10^*^ | 0.04 |  |  | -0.11^*^ | 0.03 |
| 10-Most Deprived and per 1 condition increase | |  |  | -0.09^*^ | 0.04 |  |  | -0.11^*^ | 0.03 |
| Likelihood ratio test | | *X*^2^(9) = 10.18, p=0.34 | | | | *X*^2^(9) = 17.36, p<0.05 | | | |

^*^denotes significance at p<0.05, ^**^ denotes significance at p<0.001

**Supplementary Table 12.** Cox Regression Estimates from the sensitivity analyses (to include the list of 26 conditions) of model 3 and 4 for older adults (65+ years) in England and Ontario (Canada).

|  |  | Older adults (65+ years) | | | | | | | | | |
| --- | --- | --- | --- | --- | --- | --- | --- | --- | --- | --- | --- |
|  |  | England | | | | | Ontario (Canada) | | | | |
| Covariate | Reference | Model 3 | Standard error | Model 4 | Standard error | Model 3 | | Standard error | Model 4 | Standard error |  |
| Age |  | 0.11^**^ | 0.001 | 0.11^**^ | 0.001 | 0.10^**^ | | 0.001 | 0.10^**^ | 0.001 |  |
| Women | Men | -0.26^**^ | 0.01 | -0.26^**^ | 0.01 | -0.28^**^ | | 0.014 | -0.28^**^ | 0.01 |  |
| Deprivation | 1-Least Deprived |  |  |  |  |  | |  |  |  |  |
| 2 |  | 0.09^**^ | 0.03 | 0.06 | 0.05 | -0.01 | | 0.03 | 0.05 | 0.07 |  |
| 3 |  | 0.13^**^ | 0.03 | 0.18^**^ | 0.05 | -0.01 | | 0.03 | 0.05 | 0.08 |  |
| 4 |  | 0.14^**^ | 0.03 | 0.19^**^ | 0.05 | 0.06 | | 0.03 | 0.14 | 0.07 |  |
| 5 |  | 0.14^**^ | 0.03 | 0.20^**^ | 0.05 | 0.08^*^ | | 0.03 | 0.14 | 0.08 |  |
| 6 |  | 0.17^**^ | 0.03 | 0.23^**^ | 0.05 | 0.07^*^ | | 0.03 | 0.31^**^ | 0.07 |  |
| 7 |  | 0.25^**^ | 0.03 | 0.35^**^ | 0.05 | 0.12^**^ | | 0.03 | 0.23^*^ | 0.07 |  |
| 8 |  | 0.28^**^ | 0.03 | 0.43^**^ | 0.06 | 0.11^**^ | | 0.03 | 0.24^*^ | 0.07 |  |
| 9 |  | 0.34^**^ | 0.03 | 0.50^**^ | 0.06 | 0.18^**^ | | 0.03 | 0.39^**^ | 0.07 |  |
| 10 |  | 0.43^**^ | 0.03 | 0.62^**^ | 0.06 | 0.22^**^ | | 0.03 | 0.44^**^ | 0.07 |  |
| Baseline number of conditions | No conditions | 0.21^**^ | 0 | 0.23^**^ | 0.01 | 0.30^**^ | | 0.004 | 0.33^**^ | 0.01 |  |
| Deprivation and baseline number of conditions interaction | 1-Least Deprived and no conditions |  |  | 0.01 | 0.01 |  | |  |  |  |  |
| 2 and per 1 condition increase |  |  |  | 0.001 | 0.01 |  | |  | -0.01 | 0.02 |  |
| 3 and per 1 condition increase |  |  |  | -0.02 | 0.01 |  | |  | -0.02 | 0.02 |  |
| 4 and per 1 condition increase |  |  |  | -0.02 | 0.01 |  | |  | -0.02 | 0.02 |  |
| 5 and per 1 condition increase |  |  |  | -0.02 | 0.01 |  | |  | -0.02 | 0.02 |  |
| 6 and per 1 condition increase |  |  |  | -0.02 | 0.01 |  | |  | -0.06^*^ | 0.02 |  |
| 7 and per 1 condition increase |  |  |  | -0.03* | 0.01 |  | |  | -0.03 | 0.02 |  |
| 8 and per 1 condition increase |  |  |  | -0.05* | 0.02 |  | |  | -0.04^*^ | 0.02 |  |
| 9 and per 1 condition increase |  |  |  | -0.05* | 0.02 |  | |  | -0.06^*^ | 0.02 |  |
| 10-Most Deprived and per 1 condition increase |  |  |  | -0.06** | 0.02 |  | |  | -0.06^*^ | 0.02 |  |
|  |  | *X*^2^(9) = 35.94, p<0.001 | | | | | *X*^2^(9) = 27.51, p<0.001 | | | | |

^*^denotes significance at p<0.05, ^**^ denotes significance at p<0.001
