# Supplementary material for: Socioeconomic gradient in mortality of working age and older adults with multiple long-term conditions in England and Ontario, Canada
Source: BMC Public Health. 2023 Mar 11;23:472. doi: 10.1186/s12889-023-15370-y (PMC10008074; doi:10.1186/s12889-023-15370-y)
Supplement: Supplementary file 2 — Additional file 2: Supplementary Figure 1. Linear association between number of long-term conditions and mortality for England and Ontario (Canada). Supplementary Figure 2. Distribution of number of conditions by age group and jurisdiction. [file 12889_2023_15370_MOESM2_ESM.docx]

**Additional file 2 legend:**

**Supplementary Figure 1**. Linear association between number of long-term conditions and mortality for England and Ontario (Canada).

**Supplementary Figure 2.** Distribution of number of conditions by age group and jurisdiction.

**Supplementary Figure 1**. Linear association between number of long-term conditions and mortality for England and Ontario (Canada).


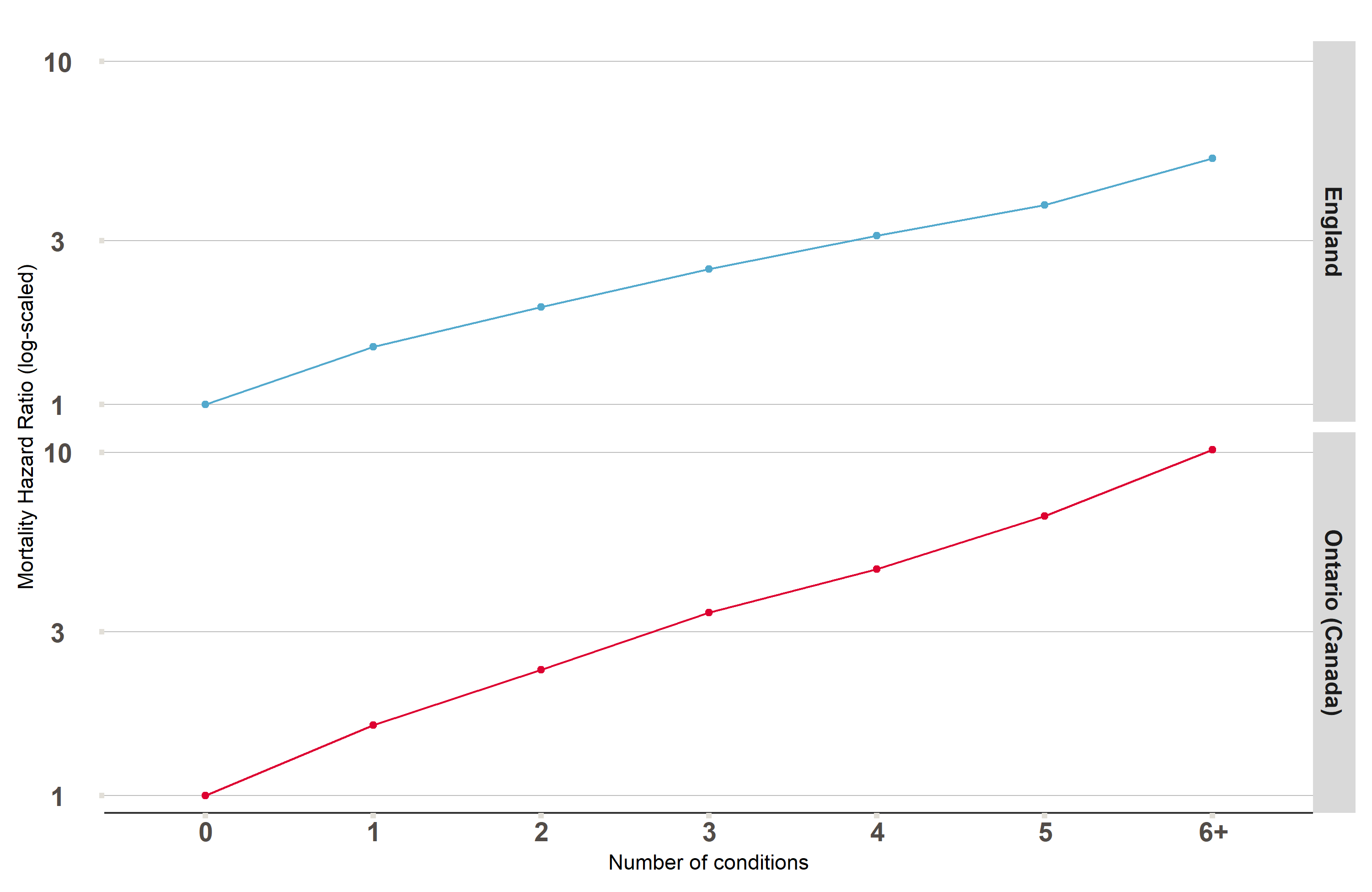


Figure shows mortality hazard ratio for death during follow-up by number of long-term conditions at baseline with 0 conditions as the reference group.

**Supplementary Figure 2.** Distribution of number of conditions by age group and jurisdiction**
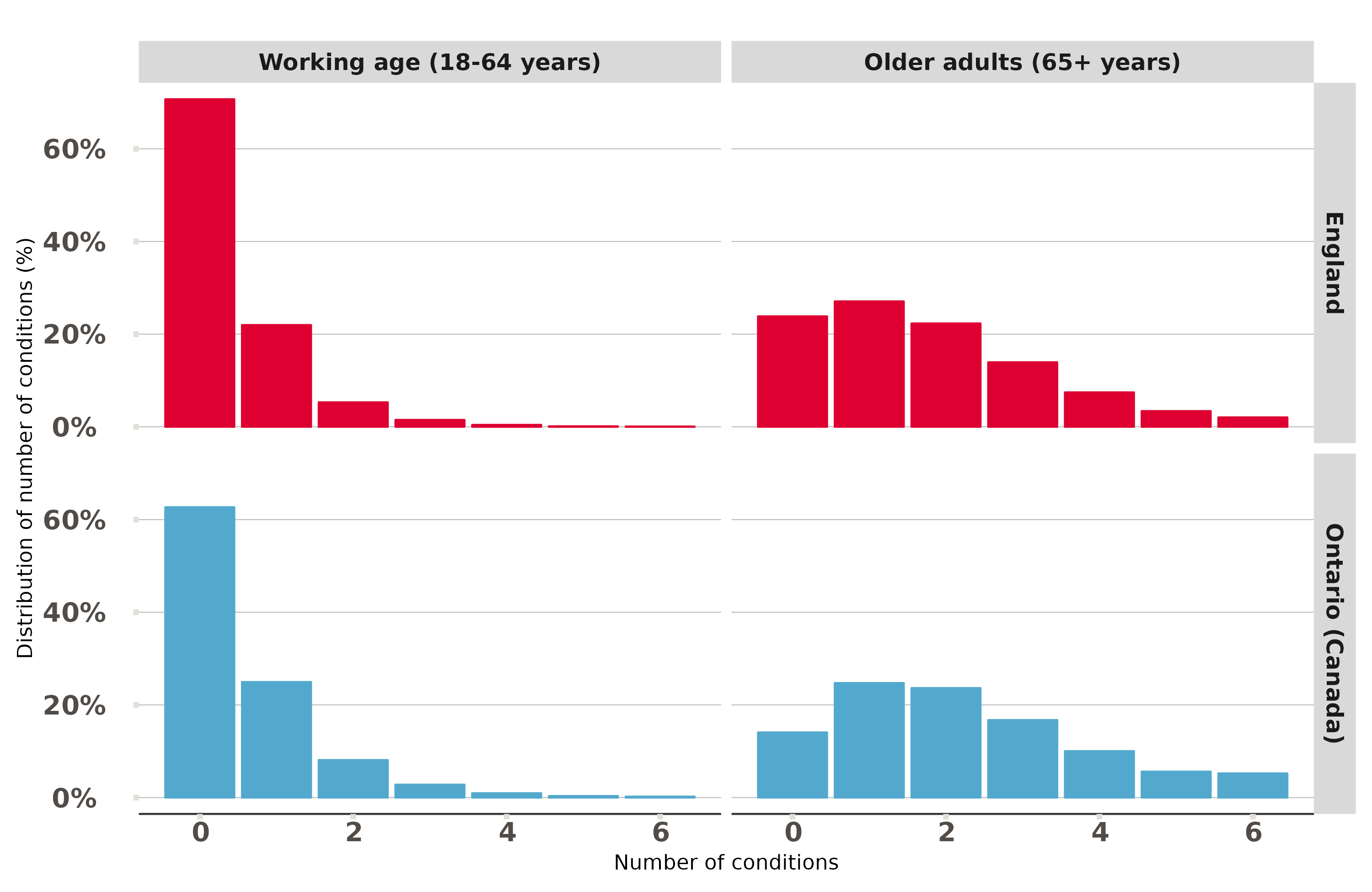
**.

Figure shows similarities in distribution of counts of conditions in working age and older adults in England and Ontario (Canada).
